# Perceptions of rural and urban residents in Borana pastoral and agro-pastoral areas in Ethiopia related to milk adulteration

**DOI:** 10.1186/s40795-024-00876-4

**Published:** 2024-04-30

**Authors:** Kebede Amenu, Abagena Shibiru, Adem Kumbe, Silvenus Ochieng Konyole, Megarsa Bedasa Jaleta, Waktole Tiki

**Affiliations:** 1 College of Veterinary Medicine and Agriculture, Addis Ababa University, Bishoftu, Ethiopia; 2 Present Address: Elweya Pastoral Development Office, Borana Zone Administration, Oromia Regional State, Elweya, Ethiopia; 3 Yabello Pastoral and Dryland Agriculture Research Centre, Oromia Agricultural Research Institute, Yabello, Ethiopia; 4 Department of Nutritional Sciences, Masinde Muliro University of Science and Technology, Kakamega, Kenya; 5 Leibniz Institute for Agricultural Engineering and Bioeconomy, Potsdam, Germany; 6 Ethiopian Civil Service University (ECSU), Addis Ababa, Ethiopia; 7 Tetra Tech ARD, Addis Ababa, Ethiopia

**Keywords:** Milk adulteration, Informal milk market, Nonmilk substance, Dairy product, Health, Ethiopia

## Abstract

Milk is a nutritious food that plays a great role in the diets of a society largely dependent on livestock production. On the other hand, contaminants can enter milk naturally or intentionally, causing a negative impact on the health of consumers. Milk adulteration is a wide concern in the dairy industry in many countries, including Ethiopia, with a subsequent negative impact on its nutritive value and potentially affecting the health of consumers. This study was designed to assess the perceptions of rural and urban residents in Borana pastoral and agro-pastoral areas in Ethiopia related to milk adulteration. It was also aimed at identifying the potential reasons for milk adulteration in the area. A semi-structured questionnaire and focus group discussions (FGDs) were used to collect quantitative and qualitative data, respectively, focusing on the types of substances added to milk and the reasons for the addition of the substances. In rural and urban areas, 73.1% and 91.7% of respondents reported suspicion of the addition of nonmilk substances or milk of other animal species to cow’s milk before selling, respectively. According to the qualitative data, most reported adulterants were water and ‘pasta or rice water’ (a murky fluid left after boiling rice or pasta). Respondents mentioned that they identify adulterated milk by observation or tasting. Economic gain was the primary perceived reason to adulterate the milk according to the study participants. The respondents had concerns about the quality and safety of milk associated with adulteration in the area. The weak enforcement of regulations related to milk quality and marketing as well as the inadequacy of capacity for the detection of adulteration were mentioned as gaps toward mitigating the problems. Awareness creation about the negative impacts of milk adulteration among the community supported by strategies for regulation, such as improving regular testing of milk and taking actions on adulterated milk, is recommended to tackle consumer concerns around milk adulteration in the area.

## Introduction

Milk is a highly nutritious food and is commonly consumed by people, but at the same time, it is prone to microbial and chemical contamination, risking the health of consumers [1, 2]. Milk adulteration is the deliberate corrupting of milk quality by the addition of nonmilk substances or milk of inferior quality [3]. The ultimate intention of milk adulteration is often to earn more benefits from increasing the volume or changing the composition [1, 4]. Adulteration can decrease the quality and safety of milk by influencing the physicochemical and microbiological properties and ultimately makes milk unsuitable for further processing or consumption [5]. The problem of food adulteration in general has been largely controlled and reduced in most developed countries through the application of very strict regulatory activities. However, food adulteration is a rampant problem in most developing countries due to the inadequacy of regulatory frameworks in the food marketing system [6].

The majority of milk produced in Ethiopia reaches consumers by passing through informal channels and through unorganized ways [7]. Such informal and minimally regulated marketing channels can expose milk and milk products to adulteration. Apart from the proneness of milk and milk products to adulteration, studies assessing such conditions are very limited in Ethiopia, with the exception of two recent studies that extensively assessed the adulteration of butter in central and southwestern Ethiopia [8] and investigated adulterants and quality of milk in urban and peri-urban areas of mixed livestock production systems [9]. The first study assessed both the perception of people about butter adulteration in central and western Ethiopia which found 94% are aware and various substances such as hydrogenated vegetable oils, Irish potato puree, banana pulps, melted tallow, wheat and maize dough, and buttermilk are added as adulterants [8]. The second study assessed the milk composition and adulterants which the results show water as common adulterant and out of 16 raw milk samples in four of them, formalin was detected. Except for the above papers, studies focusing on milk adulteration are lacking specifically in pastoral areas where milk is commonly part of the diet of the community. Assessment of the extent and reasons for adulteration can be useful inputs for designing strategies related to minimizing the problems. Hence, the present study aimed to assess the perceptions of rural and urban residents of pastoral and agro-pastoral areas of Borana regarding milk adulteration and the types of substances added as adulterants and to identify the potential reasons for the practices of milk adulteration.

## Materials and methods

### Study area

**Fig. 1:**
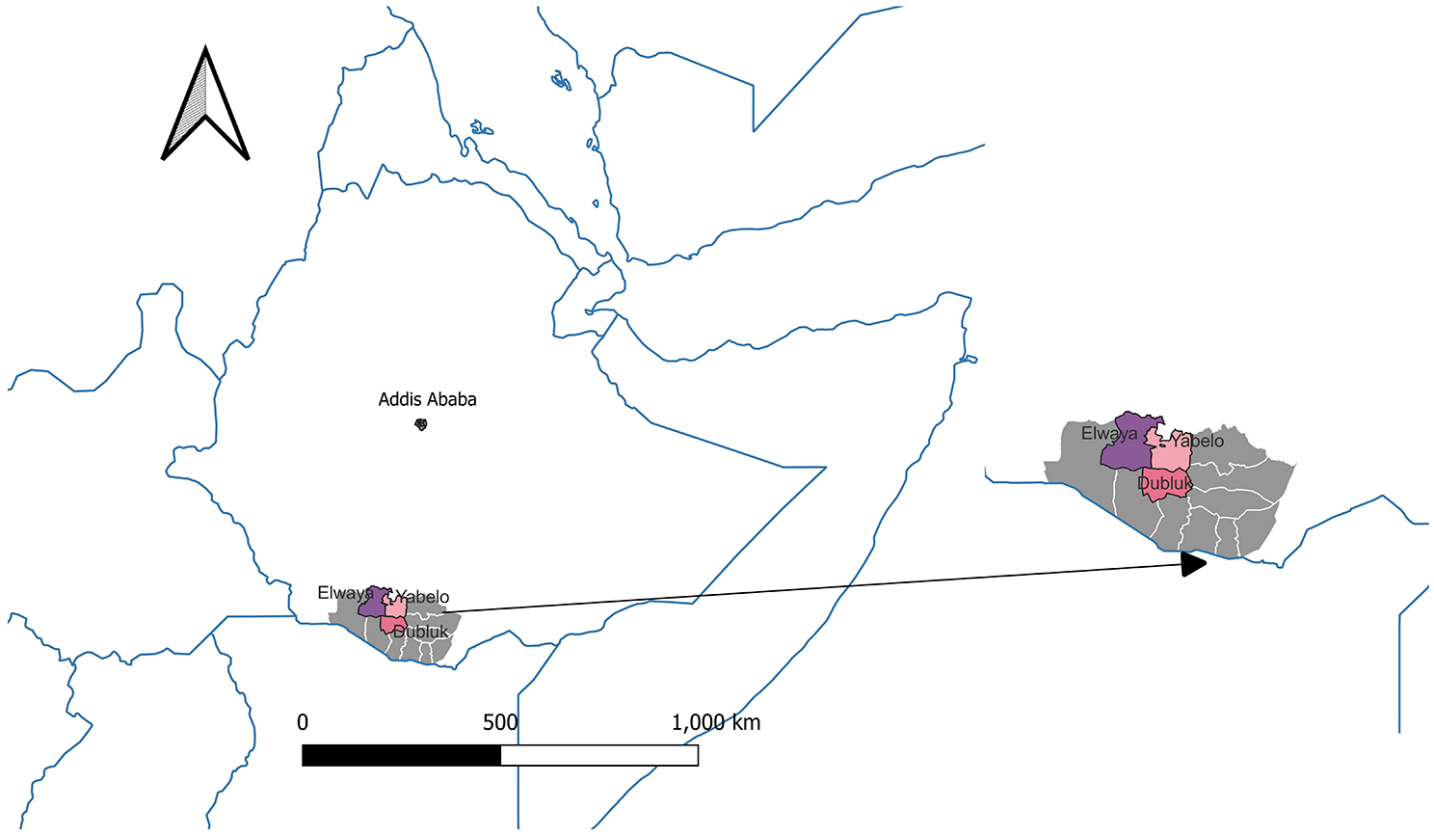
Map of the study area

The study area is located about 600 km south of Addis Ababa, capital city of Ethiopia (Fig. 1). The study was carried out in three districts (Yabelo, Dubuluk and Elweya) of the Borana Zone of Oromia Regional State, involving both urban and rural residents (Fig. 1). The area is generally characterized by a semi-arid climate with bimodal rain distribution. From the annual averages 550 to 700 mm rainfall, the area typically receives about 60% from March to May (long rainy season) and 30% from September to November (short rainy season). June to August is typically cool dry season and December to February is warm dry season. The vegetation of the area is mixed savanna with grasses and woody plants [10].

Borana pastoralists are indigenous communities living in the southern part of Ethiopia, and they are known for keeping livestock for their livelihoods [11] with limited diversified livelihoods [12]. The livestock population of Borana zone is estimated at 602,593 cattle, 420,512 sheep, 657,479 goats, 65,075 donkeys and 88,174 camels [13]. Cow milk is the most commonly marketed and consumed in the area, but camel milk is nowadays becoming common especially during the dry season of the year. Goat milk is also consumed in the area but not marketed. Milk and milk products are important components of people’s diets in the area. Selling milk or milk products was considered taboo in the past, but currently, it is an emerging source of income, especially for women [14]. Raw milk is widely consumed in Borana [15].

### Data collection methods

Mixed-method approaches involving a semi-structured questionnaire and focus group discussions (FGDs) were used for the data collection. The questionnaire was administered face-to-face involving 240 respondents (120 in rural and 120 in urban areas) selected through transect walking in the settlement areas to gather quantitative data regarding milk adulteration. Normally, the respondent has to be more than 18 years and a permanent member of the selected household through a transect walk. Four FGDs involving 8–10 participants in each session were held to collect qualitative data on the types of substances added to milk and the reasons for the additions. The data collection for the study was carried out from October to November 2018.

### Data analysis

Descriptive statistics (percentage) were calculated for data generated through a questionnaire. For the qualitative data, a narrative presentation of the views of the participants of the FGDs was quoted and described.

## Results

### Questionnaire survey

The majority (65%) of respondents in rural areas were female, while half of the respondents in urban areas were female. Nearly all (87.5%) of the rural residents were without formal education, and the corresponding value for urban residents was 25.8%. The major income source and livelihood of the urban respondents was based on trading (39.2%) and government employees (24.2%). On the other hand, 96.7% of the rural people were dependent on livestock keeping for their livelihood (Table 1).

**Table 1:** Demographic and livelihood characteristics of respondents in urban and rural areas in Borana, southern Ethiopia

Variables	Category	Urban (*n* = 120)		Rural (*n* = 120)	
		*n*	%	*n*	%
Gender	Male	60	50.0	42	35.0
	Female	60	50.0	78	65.0
Age (years)	<=30	77	64.2	48	40.0
	31–49	26	21.7	38	31.7
	>=50	17	14.2	34	28.3
Education level	No formal education	31	25.8	105	87.5
	Primary	27	22.5	12	10.0
	Secondary	25	20.8	2	1.7
	Technical and vocational education	22	18.3	1	0.8
	College/university	15	12.5	0	0.0
Occupation	Trader	47	39.2	4	3.3
	Government employee	29	24.2	0	0.0
	Pastoralist (livestock keeper)	23	19.2	116	96.7
	Student	11	9.2	1	0.8
	Daily laborer	2	1.7	0	0.0
	Work at home	2	1.7	0	0.0

Milk adulteration was believed to be a common practice in the area, where 73.1% of rural and 91.7% of urban participants of the study responded that ‘they suspect something nonmilk substance or milk of other species of animals is added to milk of cows’ before selling. According to the study participants, milk is adulterated by the addition of water and other substances, such as ‘rice or pasta water’ (murky water left after draining the cooking of pasta or rice). The study participants reported that they could check the adulteration of the milk based on their own experiences when processing and consuming the milk (e.g., clotting when boiling). Among the reported suspected reasons for increasing adulteration of milk were 1) economic reasons to earn more money, 2) the involvement of many middlemen in the market chains and 3) lack of any milk quality regulations (Table 2).

**Table 2:** Opinions of the sampled rural and urban residents about the practices of milk adulteration in Borana, southern Ethiopia (*n* = 240)

Environ	Practices	Yes	
		*n*	%
Rural (*n* = 120)	People add other substance to milk before selling	88	73.3
	Types of substance people may add to milk before selling*		
	Water	83	94.3
	Suspension remained after boiling ‘pasta/macaroni’	30	34.1
	Suspension remained after boiling rice	25	28.4
	Solution of table sugar	3	3.4
Urban (*n* = 120)	Have doubts on the quality of milk coming from rural areas	112	93.3
	People add other substance to milk before selling	110	91.7
	Habit of buying and using milk coming from rural area	118	98.3
	Means and sources of getting milk**		
	Based on prearranged agreement with milk sellers	52	44.1
	Buying from open market based on daily availability	59	50.0
	Get from family living in rural areas	10	8.5

*Out of the respondents mentioned people add other substance (adulterate); ** out of 118 who said they buy milk from rural areas

### Results of FGDs

#### Perception of milk adulteration practices

The participants of the FGDs indicated that milk is not commonly adulterated by the producers in the rural pastoral and agro-pastoral communities compared to the market level. According to the discussants, milk adulteration is largely implicated to be performed at market levels.

*For example, people in this town bought milk from the market, and upon boiling the milk, it formed a string or plastic-like consistency. There is no doubt that something was added to it with this type of milk. If the milk was pure (without adulteration), even if milk is in the process of changing into yogurt [*soured milk*”], it would not form such a chain; rather, it precipitates* (FGD 1).

The discussants mentioned that cow milk is the most adulterated, as it has high selling value and demand compared to the milk of other livestock (e.g., camel milk), and the supply of cow milk is highly reduced during the dry season. On the other hand, it was mentioned that camel milk is rarely adulterated, as the volume produced is relatively high throughout the year when compared with cow milk. Like the quantitative survey, the discussants mentioned that milk marketers can add different things to cow milk, which includes pasta water (murky solution after boiling pasta), pure water, camel milk (assumed to be cheaper), corn soup, and pond water after ‘purifying’ using lime or ash and sugar solution.

According to our discussants in this study, milk producers (cow owners) are not adulterating milk, as they consider it a cultural taboo that may result in misfortunes. Generally, milk adulteration, even the addition of camel milk to cow milk, is not acceptable in Borana. However, it was stated that those engaged in milk marketing are not following those norms and tend to adulterate the milk because their interests are largely to increase the volume of milk for sale.

It was mentioned that those who carry out milk adulteration, particularly retailers, do it ‘secretly’. However, there are mechanisms to identify adulterated milk. Different techniques of identifying adulterated milk were reported by the FGD participants. One method of identification was checking the consistency of the milk immediately after boiling, where adulterated milk forms a string-like elastic consistency. The other means to detect adulterated milk was dipping the tip of the finger into the milk and then checking the flow speed (rheology) and pattern from the fingertip; the faster the milk drop supposes the milk has been adulterated by water or other fluids while pure cow milk is sluggish to flow.

Pure *milk does not drop from fingertip, but milk having water added* [adulterated] *run down quickly* [FDG 4].

As the discussants indicated, milk adulteration has no season; however, it is very high during the dry season. One of the most important types of evidence for milk adulteration in the area was that even though actual milk production fluctuates based on season and animal disease patterns, milk is marketed throughout the year. For example, during the long dry season, milk production is reduced to a very limited amount due to feed and water scarcity. However, there can still be milk supply throughout the year, which leads informants to suspect the source and quality of the milk marketed during the dry seasons.

The discussants described these as… *“one thing which is very interesting that in last long dry season which even led the community to ask ‘is there even cow alive’, but people supply milk full of their containers [jerry cans] something the same with when the season is good and cows were okay. Therefore, we suspected that the traders supply milk added with something, otherwise where do they get milk even when cows struggle to survive?”* [FDG 1].

As most discussants explained, milk adulteration came into practice with the starting of selling milk in the area, which in the old days was considered a taboo. In the past selling milk was feared for affecting livestock health and productivity. It was stated that people started adding foreign substances to milk (adulteration) approximately after the fall of the *Dergue* regime in Ethiopia [post-1991]. Milk retailers use plastic jerry cans for milk collection from producers compared to the earlier times in which traditional containers were used. It was stated that jerry-cans can collect a greater volume of milk from different households than traditional milk containers. Informants say that some time ago, ‘*Abba Gadaa’* (traditional Borana leadership) cursed these plastic milk containers, which retailers used for milk transport to the market due to being inappropriate for local cleaning methods. The locals prefer traditional milk containers that can be easily cleansed by the traditional smoking method.

### Perceived health impacts of adulterated milk on consumers

The respondents mentioned that consuming adulterated milk has potential health impacts largely related to the signs and symptoms of illness on consumers, particularly children, which include diarrhea, vomiting and stomach discomfort or stomachache. These problems also add to the medication expenses of the family. One respondent in group [FDG 1] described the problem as follows:

‘*Milk diluted with water has no problem other than taste change, but milk added with pasta water causes stomachache and diarrhea. In addition, it causes vomiting in children*. Another discussant confirmed this by saying …. ‘*This causes a lot of illnesses to children such as diarrhea, vomiting, poor body condition which often complain; so, taking to clinic incurs another cost*. *Hence, most educated people in the town use packed or powdered milk from shops* [FDG 1].

Additionally, the discussants described that when people intentionally consume a mix of camel and cow milk, they are not happy due to changes in taste.

### Sources of milk and the value chain with factors aggravating milk adulteration

**Fig. 2:**
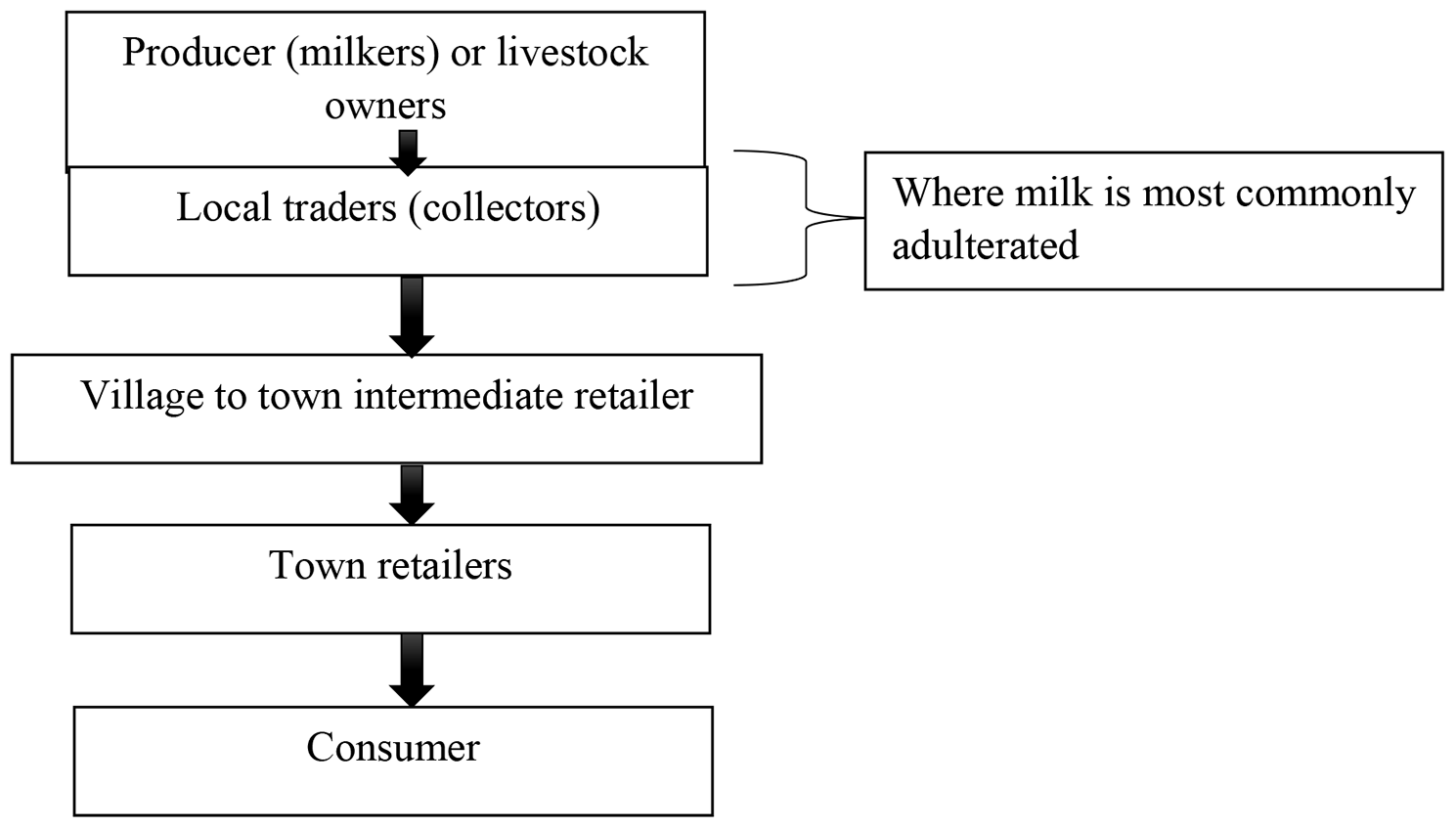
Simplified flow diagram of informal milk marketing channels in Borana

It was discussed that the marketed milk in the urban areas is derived from villagers and pastoralists. As they said, milk goes through three different stages before reaching consumers (Fig. 2). The main actors are producers, village-level milk collectors, intermediate traders, retailers at markets in urban areas and consumers. According to the participants of the FGDs, along the milk value chain the most points of adulteration are indicated in Fig. 2 below. Local traders collecting milk from rural areas (pastoralists) were indicated as main actor in the potential adulteration of milk and milk products.

It was stated that producers themselves cannot bring milk to market for sale because they are very busy with home activities, childcare, and animal rearing. Therefore, the best option is to sell to local retailers. In addition, for most of them, the marketplace is also far from their living home. Consumers prefer pure milk, even at expensive prices, over adulterated milk at a cheap price. Adulteration of milk is still not affecting the business of the sellers, as milk production is decreasing and demand surging with no other option to feed children.

Urban residents depend on the milk brought from rural villages due to the difficulty of keeping their own cows. Lack of animal feed is a constraint to urban residents to keep cows for their own milk production. Milk bought from the market is most used for children. Because milk production progressively decreases, the accessibility of milk for adults to drink is limited. As the respondents explained, the agreement regarding milk quality is mostly based on trustship. However, retailers do not respect social norms; therefore, nothing forbids them from adulterating milk. Consumers do not know where and when people have adulterated milk. Hence, it is difficult for consumers to complain formally to local administration or regulators related to the practice of milk adulteration, as it is impossible to target them due to the absence of a fixed contact address of the sellers, and most of them are not regular suppliers. There is also no legal or cultural rule to raise complaints and ask milk sellers/retailers when they suspect milk adulteration rather than leaving to buy.

## Discussion

The present study described the practices and drivers of raw milk adulteration in Borana pastoral and agro-pastoral areas by applying mixed quantitative and qualitative data collection tools. The sociocultural aspects were emphasized in the study. Borana people respect social norms by giving first place to cultural values. They give great respect and care for their animals and animal products because they are their livelihood; hence, they usually abide by social norms. Accordingly, any practices that are intentional or unintentional and can potentially harm the public or individuals (e.g., milk adulteration) are considered bad by the Borana people. Even selling milk was not a normal practice for the pastoral community, let alone adding nonmilk for economic gain. However, in the recent past, people began to breach some of those norms. For example, people began to add nonmilk products that respondents characterized as “*Hamoo*” [mean adulterant] to milk. Milk can be adulterated at different points, including at farms, at collection centers and while transporting [16]. A study in Ethiopia showed that a higher proportion of adulteration occurs at farms and collection centers in central Ethiopia [17]. However, in our present study, producers were not implicated in performing milk adulteration.

Our findings in which the common reason for milk adulteration is to increase the milk volume for economic gain are in line with several previous reports [4, 18]. As most of the discussants and the respondents in the pastoral area described, the practices of milk adulteration were for gaining more profit by increasing the volume of milk. There was no regulatory provision or formal channels of monitoring the system of milk marketing in Borana. The literature elsewhere also showed that a lack of monitoring and law enforcement with regard to food regulation is very common in developing countries [19]. Although there are many studies covering the analysis of milk adulteration in developed countries [20], the lack of accessible detection techniques makes it difficult to address and control the adulteration of foods in developing countries [19]. In the current study, customers attempt to identify milk adulteration by using their sense organs. Informal channels of milk distribution and marketing can be contributing factors for milk adulteration in developing countries [1, 4, 21].

The adulteration of milk may cause significant problems for human health related to foodborne diseases and food poisoning [5]. Chemical adulterants of milk have serious adverse health effects resulting in food poisoning, while milk adulterated with foreign proteins, such as soy, rice and almond proteins, are clinically recognized as allergens [19] and potentially compromise the nutritional content and quality of milk [6]. Adulteration causes milk to have a relatively poorer nutritional composition than normal milk. In the current study, a solution that remained after boiling pasta and rice was found to be the most commonly known adulterant added to milk, as mentioned by the study participants, and the sequelae related to having such milk were nausea, abdominal discomfort, stomachache, diarrhea, and vomiting. Confirming the claims about the health consequences was not within the scope of the present study. Milk adulteration can be another problem in addition to unhygienic practices of milk production, handling and consumption in the area [15, 22].

In the current study, the respondents described that milk is adulterated mostly at the level of traders (milk collectors from rural areas), and the result is similar to other studies, which showed that milk directly taken from producers had higher nutritional quality than milk bought from retailers with suspected adulteration [23]. In terms of the microbiological quality and physicochemical properties of milk, in Borana, milk samples obtained from household milk producers were within the acceptable standard levels and had better qualities than milk samples collected from the market [24]. In this case, for issues specific to milk adulteration traders must be targeted when interventions tackle the problems are needed.

In the present study, there was no attempt to chemically confirm or measure the types of substances added to the milk. The lack of proper facilities to detect adulterants, especially hazardous chemicals, in milk in Ethiopia, similar to many other developing countries, is a barrier to control and regulation [1, 18, 25]. Water is the most common adulterant added to milk to increase the volume of milk, and in addition to decreasing the nutritive quality of milk, it can also affect the microbiological safety and quality, especially when the added water is of poor quality [4]. Milk adulterated with contaminated water can be a serious health concern for consumers related to waterborne diseases [6]. Water for domestic use in general is of lower quality in most rural areas of Ethiopia [26, 27]. In some cases, the most protected and assumed to be safe water sources can be contaminated with *E. coli*, which is an indicator of lower microbiological quality of milk. Milk is also adulterated by mixing lower-value milk with higher-value milk. For example, in the current study, as study participants described, cow milk is adulterated with camel milk for greater profit. This is because camel milk is assumed to be ‘inferior valued’ and cheaper than cow milk. Despite the fact that health hazards related to mixing lower-valued milk are not well defined, the ethical aspect is of great concern [4]. In Borana, there is also some practice of adding milk powder to fresh cow milk as an adulterant after diluting it with more water for economic advantage.

### Limitation of the study

The present study assessed the perception of urban and rural respondents related to milk adulteration based on questionnaire survey and FGDs. It would have been best if the types of materials and the suspected adulterated products were analyzed in the laboratory. This was not possible due to lack of facility to carry out the analysis at the time of the study.

## Conclusion

The emerging marketing of milk can be considered a good opportunity for the pastoral community, but at the same time, reports of adulteration to gain unlawful income by deceitful collectors or retailers can be quite worrisome and can be a health danger. The addition of water can reduce the nutritional content of milk and can also carry bacteria, increasing the health risk. Other substances can also bring illness, but more than anything represents fraud that may make the sector lose credibility, with an impact on the income of milk value chain actors. The absence of milk quality regulations, formal milk marketing systems, and methods of adulteration detection are the major constraints for the persistence of the problem in Borana. Advocacy and awareness creation supported by designing a strategy for regulation are needed to tackle such rampant problems of milk adulteration in the area.

## Data Availability

The data can be obtained from the corresponding author upon request.
